# Bronchospasm in obese patients undergoing elective laparoscopic surgery under general anesthesia

**DOI:** 10.1186/s40064-016-2054-3

**Published:** 2016-04-12

**Authors:** Vassilios Tassoudis, Hronis Ieropoulos, Menelaos Karanikolas, George Vretzakis, Aik Bouzia, Elias Mantoudis, Argyro Petsiti

**Affiliations:** Department of Anesthesiology, University of Larissa, Larissa, Greece; Department of Anesthesiology, Washington University School of Medicine, St. Louis, MO USA; Intensive Care Unit, University Hospital of Larissa, Larissa, Greece

**Keywords:** Obesity, Bronchospasm, Laparoscopic surgery, Bariatric surgery, Airway, Oxygenation, Airway, Complications

## Abstract

**Background:**

Existing data suggest that obesity correlates with airway hyper-reactivity. However, the incidence of bronchospasm during bariatric surgery in obese patients has not been well studied.

**Methods:**

This was a prospective observational study comparing 50 obese versus 50 non obese patients undergoing elective laparoscopic surgery over a 2 year period. Bronchospasm was detected clinically by auscultation and was confirmed by measuring peak airway pressure during mechanical ventilation. Blood gases were measured at predetermined time intervals intraoperatively. Categorical variables were analyzed using Fisher’s exact test, while numerical variables within and between groups were compared using repeated measures general linear model.

**Results:**

The incidence of bronchospasm was significantly higher in obese compared to non obese patients (P = 0.027). Peak airway pressures and blood gases differed significantly when comparing non obese patients versus obese patients without bronchospasm versus obese patients with bronchospasm. Hypoventilation resulting in gradual increase of arterial PaCO_2_ was noted in all groups during surgery.

**Conclusion:**

The incidence of bronchospasm is higher in obese patients compared to non obese patients undergoing elective laparoscopic surgery. Airway pressures and blood gas values in obese patients are somewhere between values in non obese patients and values in patients with bronchospasm, thereby implying that obesity is associated with a state where bronchial smooth muscles are not fully relaxed. Consideration of increased airway reactivity in obese patients undergoing laparoscopic surgery is important for improved patient care and uneventful anesthetic course.

## Background

Published clinical data show that obesity is a risk factor for development of bronchial asthma (Camargo et al. 1999; Guerra et al. 2004; Kim and Camargo 2003; Luder et al. 2004; Schachter et al. 2001; Shore and Fredberg 2005; Weiss and Shore 2004). Seventy-five per cent of patients visiting emergency departments for bronchial asthma crisis are obese (Thomson et al. 2003), and weight loss has been documented to decrease the rate and severity of symptoms of bronchial asthma in obese patients (Aaron et al. 2004). Surgical interventions that result in weight loss in obese patients are associated with improved airway function (Macgregor and Rand 1993; Dixon et al. 1999), and obesity is associated with small airway hyperexcitability both in animals and in humans (Litonjua et al. 2002). Three theories attempt to describe the underlying mechanism of the spasm of airway smooth muscles and its consequential airway narrowing. According to the first theory spasm is caused by static and elastic forces which are enhanced in obesity, due to small lung volumes and reduced functional residual capacity (FRC) (Yap et al. 1995; Damia et al. 1988; Pelosi et al. 1997; Ding et al. 1987; Fredberg 2000; Gump et al. 2001; Sampson and Grassino 1983). However, this argument has been questioned by experimental data showing increased airway responsiveness resulting in bronchospasm in obese compared with lean mice, even when the mechanical load from excessive adipose tissue is eliminated by opening the chest wall and the lungs are exposed to ozone (Shore et al. 2003). The second explanation concerns anatomic differences leading to different lung growth (remodeling) between obese and normal children (Shore and Fredberg 2005; Aaron et al. 2004; Shore et al. 2003). Nevertheless, not all obese people were obese during childhood. The third theory implicates an inflammatory microenvironment that promotes airway narrowing in response to adipocyte-derived factors causing inflammation and bronchial irritation (Rajala and Scherer 2003; Hotamisligil 2003; Nawrocki and Scherer 2004; Chen et al. 2003).

Published data show that the incidence of postoperative complications is higher in obese surgical patients, and these complications range from minor complications, such as vomiting to major complications, such as myocardial ischemia and long term complications, such as wound disruption (Watcha and White 1992; Shenkman et al. 1993; Herrara et al. 1999). The risk of intraoperative respiratory complications, such as hypoxemia, is higher in obese adult patients (Hofer et al. 2008; Duncan et al. 1992; Chung et al. 1999) and in obese pediatric patients (El-Metainy et al. 2011) compared to non-obese patients. However, despite these concerns, obese patients can safely have ambulatory surgery (Hofer et al. 2008) and, with appropriate care and preventive measures, the number and severity of complications is low (Choban and Flancbaum 1997; Dindo et al. 2003; Thomas et al. 1997).

Based on the above observations, we designed a prospective observational study of adult patients undergoing elective abdominal laparoscopic surgery under general anesthesia, in an attempt to evaluate bronchospasm in obese patients undergoing laparoscopic bariatric surgery versus non-obese patients undergoing other laparoscopic general surgery procedures.

## Methods

This prospective observational study enrolled one hundred patients undergoing elective laparoscopic abdominal surgery under general anesthesia, divided in two groups: One group included 50 patients with normal to excess body weight (BMI < 35 kg/m^2^, group A) who underwent elective laparoscopic cholecystectomy, hernia repair or colectomy. These patients were compared with a group of 50 patients with severe to morbid obesity (BMI ≥ 35 kg/m^2^, group B) who underwent laparoscopic sleeve gastrectomy.

Patients were offered the option of participating in the study, based on timing of their surgery (“first come, first serve”). Enrollment in each group continued until enough patients consented to reach the predetermined goal of enrolling 50 patients per group. This was a prospective observational study, and therefore there was no randomization and no “blinding” issues. The study was approved by the Institution Ethics Committee, was conducted at a tertiary care University Hospital over a 2 year period, and was registered at the “Clinical Trials” international trial registry (ClinicalTrials.gov Identifier: NCT01488643).

Sample size calculation was conducted before the study started, using the G*Power Version 3.1.9 sample size calculation program, which is freely available from the University of Dusseldorf, in Germany. Sample size calculation was based on the following assumptions: Primary outcome is the presence of bronchospasm, and expected bronchospasm incidence is 15 % in obese patients versus 0.5 % in healthy non-obese patients. In obese patients, the incidence of asthma has been reported in a range from as low as 10–12 % (Beuther and Sutherland 2007; Luder et al. 2004) to as high as 35 % (Schachter et al. 2001). Therefore, we believe that bronchospasm incidence of 15 %, which is the basis of our power analysis is reasonable, because it is supported not only by our unpublished data, but also by earlier published studies. Based on these assumptions, sample size calculation showed that, for alpha error = 0.05, our study would need 48 patients (rounded to 50 patients) per group in order to have beta error = 0.2, therefore for the study to have power = 1 − b = 0.8.

Initial evaluation of obese patients for inclusion in the study was done at their first scheduled preoperative assessment. Inclusion criteria were written informed consent and age > 18 years. Exclusion criteria were history of psychiatric disease or mental disorder, use of marijuana or other habit-forming drugs and inability to follow preoperative orders. All patients were instructed to quit smoking at least 8 weeks before scheduled surgery. On admission to the hospital, patients were asked if they followed preoperative instructions and quit smoking before their operation (Warner 2006). All patients stated they quit smoking at least 2 months (=8 weeks) before surgery. All patients were evaluated with standardized anesthetic preoperative assessment (The Association of Anaesthetists of Great Britain and Ireland 2007). This was important because morbidly obese patients can have profound cardio-respiratory dysfunction which remains asymptomatic due to limited mobility. Preoperative assessment also included screening for obstructive sleep apnea (OSA), which is common in morbidly obese patients and can predispose to hypoventilation, difficult mask ventilation, shunt with rapid arterial oxygen desaturation. In addition, OSA warrants particular attention during emergence from anesthesia, extubation and in the immediate postoperative period.

In addition, when pre-anesthesia evaluation raised concerns for potential difficult airway, patients were also evaluated by ENT (Ear Nose and Throat) surgeon using flexible endoscopy to assess airway patency and potential for difficult mask ventilation and/or endotracheal intubation. Demographic and clinical data, including age, sex, height, weight, body mass index (BMI), ASA physical status, tobacco use and comorbidities, including hypertension, asthma, chronic obstructive pulmonary disease (COPD) and OSA were collected and stored in a secure encrypted computer. Data were collected by physicians trained in data collection, and were immediately reviewed by a senior investigator to confirm data completeness, consistency and reliability.

In the operating room monitoring included five lead ECG and continuous arterial blood pressure monitoring after insertion of radial arterial line. All study participants were informed about placement of a radial arterial line just for the purposes of this study. Allen’s test was performed in all patients before proceeding with radial artery catheterization.

Anesthesia was induced with intravenous (IV) midazolam 2 mg, propofol 3 mg/kg and fentanyl 150 mcg. After ability to manually ventilate was confirmed, cisatracurium 0.2 mg/kg was given to facilitate endotracheal intubation. All medication doses were calculated based on ideal body weight. Anesthesia was maintained with sevoflurane at 1.2 end-tidal MAC (corrected for age) combined with fentanyl and remifentanil.

The Drager Primus anesthetic workstation (Drager, Inc, Lubeck, Germany) was used for all cases. This workstation has a sophisticated ventilator that can provide different modes of ventilation, including volume control and pressure control ventilation. Mechanical ventilation was standardized using the following settings: Volume Control mode, tidal volume 6 ml/kg based on ideal body weight, inspiratory O_2_ fraction (FiO_2_) 0.5, positive end expiratory pressure (PEEP) 5 cm H_2_O, inspiratory to expiratory ratio 1:2 with 10 % plateau, and target P_max_ ≤ 30 cm H_2_O. Muscle relaxation was monitored with quantitative TOF-determination (TOF Watch; Organon, Dublin, Ireland), whereby TOF ratio <0.9 indicated reversal of neuromuscular blockade. Pneumoperitoneum pressure was up to 12–13 mmHg, in order to maintain consistent intraoperative intraabdominal pressure in all patients. Multimodal analgesia was initiated 20 min before the end of surgery with combination of IV morphine 5 mg and IV paracetamol 1 gm. Postoperative analgesia included tramadol 100 mg IV twice a day, paracetamol 600 mg IV four times a day and rescue morphine 2–5 mg IV for 3 days.

In order to standardize diagnosis and treatment during the study, the diagnosis of bronchospasm was based on intense wheezing detected by auscultation. Other signs supporting the diagnosis of bronchospasm were tidal volume reduction (hypoventilation), increased circuit pressure and prolonged expiration with visible upslope on the capnogram due to increased airway resistance, arterial blood oxygen desaturation and hypoventilation. End-tidal CO_2_ values measured with waveform capnography are not reliable for diagnosis of bronchospasm, because they can rise due to hypoventilation, but can also fall in cases of severe bronchospasm due to insufficient gas exchange. Therefore, we obtained repeated arterial blood gas samples in order to measure PaCO_2_ and PaO_2_ as markers of hypoventilation and arterial blood desaturation, rather than rely solely on expired gas measurement.

The study protocol instructed the anesthesiologist to start treatment with bronchodilators as soon as bronchospasm was confirmed. The protocol also suggested avoiding airway irritation due to extensive manipulation, light anesthesia, endotracheal tube misplacement, aspiration or other stimuli that could lead to bronchial irritation and spasm. In cases where bronchospasm was suspected, the anesthesiologist was instructed to (a) check the endotracheal tube for narrowing/obstruction by kinking or secretions, (b) look for rashes that could be manifestation of allergic reaction, (c) check for cyanosis or desaturation to ensure that oximetry signals are valid, and (d) attempt to ventilate manually while listening for breath sounds and observing airway pressures. Peak and plateau pressures were measured together to help detect what the problem was: elevation of both peak and plateau pressures is more likely caused by intrathoracic cause (bronchospasm), whereas isolated peak airway pressure increase without change in plateau pressure could indicate a problem at or proximal to the ETT, such as mechanical tube narrowing (Stenqvist et al. 1979). If bronchospasm was detected, treatment options included increasing FiO_2_ to 100 % to improve oxygenation, b2-receptor agonists or b1, b2 mixed agonists, corticosteroids and methyl xanthenes in order to achieve bronchodilation.

Peak pressure and arterial blood gas values were recorded at pre-defined time points throughout the perioperative period. During pneumoperitoneum, data were collected 10, 30 and 50 min after onset of the pneumoperitoneum, and these time points are reported as T1, T2 and T3, respectively. Data were analyzed using the SPSS v.17 statistical software package (SPSS Inc., Chicago, IL). For analysis purposes, patients were divided in 2 groups, based on whether they had (1) or not (0) a characteristic for each independent variable. Groups were assigned to obese patients (1), non obese (0), and to patients with (1) or without bronchospasm (0). Categorical variables were compared using Chi square or Fisher’s exact test, as appropriate. Repeated Measures General Linear Model analysis was used to detect statistical differences between groups and within groups at different time points. Observed differences were considered significant when P < 0.05.

## Results

In total, our study enrolled 100 patients (54 women, 46 men), and mean age was 44.5 years. Patients were allocated into two groups (50 obese patients vs. 50 non obese patients) using BMI value of 35 kg/m^2^ as the cut-off point. Mean BMI was 46.32 ± 6.97 kg/m^2^ (morbid obesity ≥40 kg/m^2^) in obese patients versus 28.34 ± 3.31 kg/m^2^ in non obese patients. Mean ASA value was 3.16 ± 0.37 in obese versus 2.01 ± 0.57 in non obese patients. There was no significant difference between the groups with regards to cigarette smoking (P = 0.671) or hypertension (P = 0.833). In the obesity group, 2 patients had known bronchial asthma (ns) and 24 patients had known OSA (P < 0.001). All patients completed the study, there were no cases with missing data, and the results are summarized in Table 1.

**Table 1:** Demographic and clinical patient characteristics

	Obese (n = 50)	Non obese (n = 50)	P
Males/females	16/34	30/20	0.009
Age (years)	40.96 ± 9.67	48 ± 16.89	0.012
ASA status	3.16 ± 0.37	2.01 ± 0.57	0.000
Weight (kg)	132.44 ± 17.82	79.88 ± 6.59	0.000
BMI (kg/m^2^)	46.322 ± 6.976	28.347 ± 3.31	0.000
Smoking	18	15	NS
Bronchial asthma	2	0	NS
Obstructive sleep apnea	24	0	0.000
Hypertension	16	18	NS

Continuous variables are reported as Mean ± SD. Values are compared using *t* test, Chi square test or Fisher’s exact test as appropriate

**Fig. 1:**
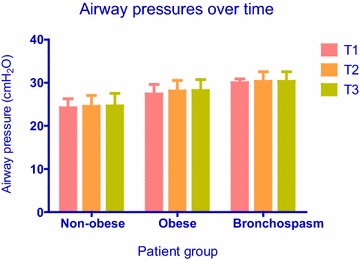
Airway pressure values (Mean, SD) over time in non-obese patients versus obese patients without bronchospasm versus obese patients with bronchospasm. Between groups comparisons showed significantly (P = 0.000) higher airway pressures in obese compared to non-obese patients, and significantly (P = 0.01) higher airway pressures in patients with bronchospasm compared to patients without bronchospasm at all time points. Within groups comparisons did not show significant changes of airway pressures over time (P = 0.08)

**Fig. 2:**
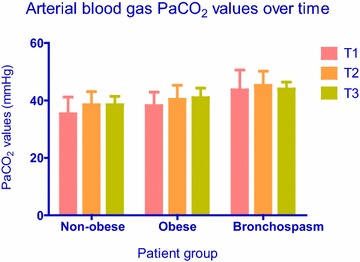
Arterial blood gas PaCO_2_ values (Mean, SD) in non-obese patients versus obese patients without bronchospasm versus obese patients with bronchospasm over time. Between groups comparisons showed significantly (P = 0.005) higher PaCO_2_ values in obese compared to non-obese patients, and significantly (P = 0.012) higher PaCO_2_ values in patients with bronchospasm compared to patients without bronchospasm at all time points. Within groups comparisons showed significant (P = 0.015) change of PaCO_2_ values over time, as seen in the graph

**Fig. 3:**
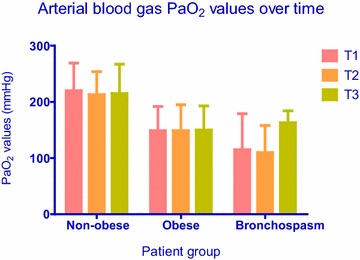
Arterial blood gas PaO_2_ values (Mean, SD) in non-obese patients versus obese patients without bronchospasm versus obese patients with bronchospasm over time. Between groups comparisons showed significantly (P = 0.000) lower PaO_2_ values in obese compared to non-obese patients at all time points. Although PaO_2_ values were lower in patients with, compared to patients without bronchospasm, the difference did not reach significance (P = 0.305). Within groups comparisons showed significant (P = 0.000) change of PaO_2_ values over time, as seen in the graph

Six patients had intraoperative bronchospasm, and all six belonged in the obese group (6/50 vs. 0/50; P = 0.027 by Fisher’s exact test). These patients were then analyzed as a subgroup among patients with obesity. In all cases, bronchospasm was detected immediately after induction of anesthesia and persisted despite all treatment efforts at least until the time of the third measurement (T3) or until the end of surgery. Peak airway pressure, PaCO_2_, and PaO_2_ data at sampling times T1, T2 and T3 from non-obese patients versus obese patients without bronchospasm versus the subgroup of obese patients with bronchospasm are presented in Figs. 1, 2 and 3, respectively. With regards to peak airway pressure, comparison between groups showed gradual increase in values, denoting that peak pressure in obese patients is higher than in non-obese patients. Furthermore, peak airway pressure in obese patients with bronchospasm was higher compared to obese patients without bronchospasm (Fig. 1). PaCO_2_ measurements showed a similar pattern whereby ventilation was best in non-obese patients, lower (hypoventilation) in obese patients without bronchospasm, and even lower (worst) in obese patients with bronchospasm (Fig. 2). PaO_2_ values showed a similar pattern, with gradual decline between groups insinuating that hypoventilation and airway restriction lead to gradual arterial blood desaturation; however, the last PaO_2_ value was elevated (sampling time T3; 163 ± 21 mmHg, Fig. 3) because FiO_2_ was increased in an attempt to treat hypoxemia.

## Discussion

Our observation that bronchospasm is a clinically relevant issue in obese patients undergoing bariatric laparoscopic surgery, together with the paucity of data on the subject motivated us to conduct this prospective observational study. Our results showed high incidence of bronchospasm (6 of 50, 12 %) in obese patients, which is significantly higher compared to the incidence in non-obese patietns (0 of 50, 0 %, P = 0.027). There is no clear understanding why the incidence of bronchospasm was higher in obese patients. However, it is plausible that, because of increased bronchial excitability, common airway irritants can trigger bronchospasm in obese patients, but not necessarily in non-obese patients whose bronchial smooth muscle is not as prone to spasm.

As expected, patients developing bronchospasm showed significant differences regarding airway pressures and PaCO_2_ with hypoventilation and deterioration of arterial blood PaO_2_ values. However, because FiO_2_ was increased from 50 to 100 % in order to maintain adequate oxygenation in patients with bronchospasm, PaO_2_ values cannot support any safe conclusions with regards to oxygenation. Both variables indicate that gradual smooth muscle constriction leads to increasing airway resistance which, in turn, leads to hypoventilation and high airway pressures due to airway narrowing. Arterial blood oxygen levels followed a reverse pattern compared to airway pressures and PaCO_2_: the augmented PaO_2_ value at time point T3 was not surprising because the attending anesthesiologist increased FiO_2_ from 0.5 to 1 in order to maintain adequate arterial oxygen saturation. Our analysis did not show significant airway pressure changes over the entire surgical procedure, thereby suggesting that time in itself did not have a significant effect on airway pressures. Between groups analysis showed that obesity was a significant factor, and there were differences in blood gas and airway pressure values between obese versus non obese patients, as mentioned above. Patients with bronchospasm had significantly higher airway pressure and PaCO_2_ values compared to patients without bronchospasm. However, differences in PaO_2_ values did not reach statistical significance, because FiO_2_ adjustments aimed at maintaining adequate saturation by oximetry affected PaO_2_ values in patients with bronchospasm.

Within groups comparisons did not show a significant interaction between time and airway pressures. The observation that airway pressures did not change significantly over time suggests that bronchospasm occurred early during the case, and treatment did not result in measurable improvement. In contrast, arterial blood gas values showed significant time effect: hypoventilation occurred in all groups and persisted over time, even though ventilation was still considered satisfactory, whereas oxygenation worsened and resulted in desaturation over time only in obese patients with bronchospasm. Bariatric surgery patients may be more susceptible to hypoventilation and impaired oxygenation, because general anesthesia and pneumoperitoneum can exaggerate the respiratory dysfunction already present in morbid obesity. General anesthesia reduces FRC by up to 20 % in healthy individuals and by up to 50 % in obese patients (Bjerkedal 1957). However, we cannot attribute this effect to bronchospasm, because airway pressures did not change significantly over time. There was no obvious synergistic effect over time in obese patients with regards to airway pressure, PaCO_2_ or PaO_2_, meaning that no variable changed more over time in the obese group. As discussed earlier, because the observed synergistic effect of PaO_2_ over time in patients with bronchospasm was due to increasing FiO_2_, this finding cannot support any valid conclusions.

In our study, the obese patient group had higher ASA physical status compared to non-obese patients, but this difference was expected because of the higher number of comorbidities in obese patients, and is in agreement with earlier studies (Bjerkedal 1957; Adams and Murphy 2000). Morbidly obese patients with high proportion of visceral fat are at increased risk for cardiovascular disease, left ventricular dysfunction, hypertension and stroke, and have higher incidence of OSA. In our sample, obese patients were relatively young and there was no association between obesity and smoking or bronchial asthma, despite the fact that obesity is a possible contributor to asthma, as discussed in the introduction. It is possible that our study design and small sample size did not have adequate power to demonstrate the association between obesity, smoking and hyper reactive airways.

The use of morphine in patients with pre-existing asthma or intraoperative bronchospasm could be subject to criticism. However, published data suggest that morphine is well tolerated, and may even be beneficial in patients with asthma (Soleymani et al. 1972; Blumberg 1973; Eschenbacher et al. 1984; Rutherford et al. 2002; Otulana et al. 2004). Furthermore, because hydromorphone is not available for clinical use in Greece, morphine and meperidine were the only medium-long acting opioids available for use in our patients. We therefore chose to use morphine for analgesia because this is the opioid most frequently used in our clinical setting, and also due to concerns about the adverse effects of meperidine.

The absence of preoperative pulmonary function data is a limitation of this study. However, current guidelines, including the latest guidelines on the perioperative management of the obese surgical patient that were published in 2015 do not require preoperative pulmonary function testing in obese patients undergoing surgery (Nightingale et al. 2015).

Morbid obesity probably defines a special category of patients with unique pathophysiologic changes and increased risk of perioperative complications. Obese people are affected by several serious, potentially life threatening health issues, including respiratory morbidity which worsens during laparoscopic surgery. Compared to patients with normal-weight, obese patients have increased metabolic needs, hypervolemia due to higher extracellular volume, increased cardiac output and enlarged pulmonary vascular system (Adams and Murphy 2000). These changes can result in increased lung resistance and decreased compliance, thereby contributing to hypoventilation and arterial blood desaturation. In agreement with the literature regarding obese awake individuals (Shore and Fredberg 2005), the increased airway pressures and PaCO_2_ and decreased PaO_2_ between groups observed in our study suggest that obese patients undergoing laparoscopic surgery are in a “pre-bronchospasm state” and therefore are prone to bronchospasm. This “pre-bronchospasm state” could be the consequence of chronic lung inflammation. Elevated serum cytokines, chemokines and adipocyte derived factors, such as leptin, adiponectin and plasminogen activator inhibitor are potential factors that could result in chronic sensitization of smooth muscles, thereby altering bronchial smooth muscle function and promoting airway narrowing. These considerations emphasize the importance of careful planning of the anesthetic technique for morbidly obese patients undergoing laparoscopic surgery. Even though these are “routine operations” from the surgeon’s point of view, the anesthesiologist should be vigilant about potential problems and carefully plan the anesthetic regimen, in order to reduce the risk of perioperative respiratory or other adverse events and optimize patient well being.

In conclusion, perioperative bronchospasm is a significant issue in obese patients undergoing laparoscopic surgery. As the diagnosis of bronchospasm is based on peak airway pressure and blood gas changes, recording of baseline values is essential. Because of increased smooth muscle irritability, bariatric patients are likely to be in a “pre-bronchospasm state”, with airway pressure values somewhere between values observed in non obese patients and values observed in obese patients with known bronchospasm. More studies on large number of patients are needed to clarify the role of bronchospasm and its effect on postoperative respiratory function and outcome in obese patients undergoing laparoscopic surgery.
